# Developmental Trajectories of Positive Expectancies of Cannabis Use Effects Among Early Adolescents: Longitudinal Observational Study Using Latent Class Growth Analysis

**DOI:** 10.2196/85652

**Published:** 2026-01-09

**Authors:** Weisiyu Abraham Qin, Dong-Chul Seo, Wura Jacobs, Sijia Huang, Kit K Elam

**Affiliations:** 1 Center for Tobacco Control Research and Education, Cardiovascular Research Institute, University of California, San Francisco, 530 Parnassus Ave., Ste 366, San Francisco, CA, 94143, United States, 1 4155023083, 1 4155149345; 2 Department of Applied Health Science, School of Public Health, Indiana University Bloomington, Bloomington, IN, United States; 3 Department of Applied Psychology in Education and Research Methodology, School of Education, Indiana University Bloomington, Bloomington, IN, United States

**Keywords:** positive cannabis use expectancy, latent class growth analysis, family dynamics, early adolescents, parental monitoring, family cannabis use rules, family conflict

## Abstract

**Background:**

Positive expectancies of cannabis use effects, which are the beliefs about the anticipated positive effects of cannabis, are robust cognitive precursors of adolescent cannabis initiation and escalation. However, little is known about how sociodemographic, familial, and psychopathological factors predict positive expectancies of cannabis use effects or how these expectancies evolve across early adolescence.

**Objective:**

This study aimed to identify distinct developmental trajectories of positive expectancies of cannabis use effects among early adolescents, as well as the longitudinal effects of familial factors on positive expectancies of cannabis use effects over time.

**Methods:**

This study used latent class growth analysis with 3 waves of longitudinal data from the Adolescent Brain Cognitive Development Study (ABCD Study) to identify distinct trajectories of positive expectancies of cannabis use effects among a large, demographically diverse cohort of early adolescents (aged 10‐13 years). Multinomial logistic regression was used to examine whether baseline sociodemographic and policy-level factors were associated with class membership. Time-varying effects of familial factors (ie, parental monitoring, family cannabis use rules, and family conflict) and adolescents’ psychopathology were examined within and across trajectory classes using class-specific and common effects models.

**Results:**

Four distinct trajectories of positive expectancies of cannabis use effects emerged with different profiles: moderate-increasing (3118/7409, 42.1%), high-increasing (2111/7409, 28.5%), low-increasing (1496/7409, 20.2%), and high-decreasing (684/7409, 9.2%) trajectories. Parental monitoring and strict family cannabis use rules consistently predicted lower positive expectancies of cannabis use effects, particularly in the moderate- and high-increasing groups, while family conflict emerged as a robust risk factor. Psychopathological symptoms became increasingly predictive of positive expectancies of cannabis use effects at later ages, suggesting a developmental shift in vulnerability.

**Conclusions:**

The development of positive expectancies of cannabis use effects in early adolescence is heterogeneous and shaped by the interplay among sociodemographic, familial, and psychopathological factors. These findings highlight the critical window for early, family-based prevention and underscore the importance of tailoring intervention strategies to specific developmental and risk profiles.

## Introduction

### Background

Adolescent cannabis use is a significant public health concern in the United States. Despite its federally illegal status [1], it is estimated that 11.2% (2.9 million) of US adolescents (aged 12‐17 years) used cannabis during the past 12 months [2]. Prior research has shown that early initiation, frequent use, and escalating cannabis use during adolescence are associated with a range of adverse developmental outcomes, including academic underachievement, impaired social functioning, increased risks for depression and suicidality, elevated likelihood of developing substance use disorders, and poorer psychosocial and occupational functioning in later adulthood [3-6]. Understanding cognitive antecedents of cannabis use, particularly positive expectancies of cannabis use effects, is critical for effective prevention.

Substance use expectancies are beliefs about the anticipated effects of using a particular substance, which can serve as critical proximal cognitive mechanisms determining whether an individual will initiate the use of a substance or continue substance use later in life [7-12]. Furthermore, substance use expectancies serve as a core construct in various psychological theories explaining substance use behavior [13], including social learning theory [14-16]; expectancy theory [17]; and plans, responses, impulses, motives, and evaluations (PRIME) theory [18]. Social learning theory emphasizes that substance-related cognitions are acquired through observational learning, modeling, and reinforcement in salient social contexts, such as the family. Expectancy theory and PRIME theory expand on this by conceptualizing that expectancies form as part of a broader evaluative cognitive network that guides motivation, decision-making, and dynamic behavioral choices, which precedes and organizes actual substance use behavior.

Guided primarily by social learning theory, this study focuses on examining how early adolescents, who are particularly sensitive to familial cues, are likely to form positive expectancies of cannabis use effects in response to familial factors. In this context, defining trajectories of positive expectancies of cannabis use effects and identifying family factors (eg, household rules, parental monitoring, and family conflict) that predict membership in different trajectories of positive expectancies of cannabis use effects are essential for informing early interventions and refining theoretical models of cannabis use during early adolescence.

### Positive Expectancies of Cannabis Use Effects

Positive expectancies of cannabis use effects include anticipated feelings of relaxation, enhanced creativity, and social connection when using cannabis [19,20], and have been consistently identified as key cognitive drivers of cannabis use behaviors [21-28]. Adolescents who hold more positive beliefs about the anticipated effects of cannabis use are significantly more likely to initiate cannabis use at an earlier age and engage in sustained and/or escalating use over time, even after controlling for other known established risk factors [29,30]. This underscores the unique etiological role of positive expectancies of cannabis use effects in shaping the developmental trajectories of future cannabis initiation. More importantly, positive expectancies of cannabis use effects are modifiable, making them compelling targets for early interventions, before beliefs become firmly entrenched.

Despite growing concerns surrounding adolescent cannabis use and the need for prevention, research has largely focused on behaviors emerging in late adolescence, often neglecting early adolescence (ages 10‐14 years), a critical period when expectancies develop before direct cannabis experimentation [31,32]. During this period, there is rapid cognitive, emotional, and social development, and environmental influences play formative roles in shaping substance-related expectancies. Among these, family factors, such as parental monitoring, household rules, and family conflict, are particularly influential as they structure adolescents’ early views of substance use [33,34].

### Family Influences on Positive Expectancies of Cannabis Use Effects

Family rules regarding substance use, parental monitoring, and family conflict all have robust influences on shaping adolescents’ substance-related expectancies but have yet to be examined relative to positive expectancies of cannabis use effects. Substantial research has demonstrated that parental alcohol and tobacco rules influence alcohol and tobacco expectancies and subsequent use [35,36]. Empirical studies focusing on cannabis use have shown that clear, well-defined family rules on cannabis use serve as protective factors for cannabis use, whereas the absence or ambiguity of such rules is linked to increased cannabis use [37]. However, research has yet to examine the influence of family rules on positive expectancies of cannabis use effects. It may be that parents who clearly communicate the risks of substance use and enforce explicit household rules indirectly cultivate lower positive expectancies of cannabis use effects in their children, whereas permissive or neutral parental attitudes on substance use may promote more favorable expectancies about cannabis effects. Notably, findings from broader literature on substance use expectancies may not fully extend to positive expectancies of cannabis use effects. The distinct social, legal, and perceived medicinal aspects of cannabis use may lead adolescents to form unique expectancies that differ from those observed for alcohol or tobacco.

Parental monitoring, defined as active supervision and awareness of adolescents’ activities [38], represents another critical protective factor across various domains of adolescent risk behavior. Parental monitoring has been consistently associated with reduced alcohol, cannabis, and nicotine use across diverse demographic groups [39-42]. In addition to deterring actual use behaviors, higher levels of parental monitoring are associated with a lower intention to initiate substance use [43]. Given the demonstrated impact on behavioral intentions and decision-making, higher levels of parental monitoring may also reduce adolescents’ positive expectancies of cannabis use effects, although direct empirical evidence remains limited.

Family conflict has been robustly associated with an increased risk of substance use and more favorable expectancies of alcohol use [44-47]. Mechanistically, conflict may undermine parental authority, increase psychological distress, and elicit maladaptive coping strategies, thereby engendering positive attitudes toward substance use [48,49]. While most research has centered on alcohol, the underlying mechanisms are likely applicable to cannabis, warranting extension of these findings to positive expectancies of cannabis use effects. Thus, family rules, parental monitoring, and family conflict represent key proximal determinants of the formation and trajectory of positive expectancies of cannabis use effects. Understanding their dynamics provides important leverage points for targeted interventions that may disrupt adolescent cannabis risk trajectories.

Of note, previous studies on substance use expectancies have relied on conventional growth models to examine developmental trajectories [11,50]. These variable-centered approaches assume that all individuals within a population follow a single average growth trajectory and posit that covariates influencing growth factors affect all individuals uniformly [51]. These variable-centered approaches overlook the possibility of distinct subgroups with divergent developmental pathways, limiting the ability to capture the complexity of early adolescent development [52]. To address this limitation, we used latent class growth analysis (LCGA), a person-centered alternative that classifies individuals into distinct subgroups following similar trajectories, thereby capturing unobserved variation in adolescent development [53,54].

### Our Study

In this study, we used LCGA to identify distinct developmental trajectories of positive expectancies of cannabis use effects and examine how parental monitoring, family cannabis use rules, and family conflict are associated with trajectories of positive expectancies of cannabis use effects both within and across trajectory classes while adjusting for demographic characteristics. By integrating a person-centered, longitudinal approach, this study seeks to advance our understanding of how familial factors shape the formation and progression of positive expectancies of cannabis use effects during this critical developmental period.

## Methods

### Data and Study Sample

The data were drawn from the Adolescent Brain Cognitive Development Study (ABCD Study), the largest ongoing longitudinal investigation of development and health among early adolescents in the United States. Funded by the National Institutes of Health (NIH) and conducted across 21 research sites using a rigorous multistage sampling design, the ABCD Study provides a unique opportunity to understand the factors shaping adolescent development, substance use behaviors, and mental health outcomes [55,56]. Recruitment was carried out between 2016 and 2018 using a systematic school-based sampling approach designed to approximate the demographic composition of the national population of 9- and 10-year-old children [57]. Schools were selected through probability sampling methods stratified by geographic region, race and ethnicity distributions, and socioeconomic characteristics. ABCD Study teams coordinated with school administrators to distribute study information, conduct on-site presentations, and invite families to participate [57]. Additional information regarding study design, methodology, and data accessibility can be found on the study website [58]. The present analysis included data from the 1-year (mean age 10-11 years), 2-year (mean age 11-12 years), and 3-year (mean age 12-13 years) follow-up waves. The 1-year follow-up was designated as baseline (T1), with subsequent waves designated as time 2 (T2) and time 3 (T3) for this study.

Participants who had valid data on the outcome variables at T1 were included, yielding an initial analytic sample of 8841 participants. Between T1 and T2, 418 participants were lost to follow-up, and an additional 606 participants were lost between T2 and T3. Furthermore, 408 cases were excluded due to missing poststratification weights, which are required for population-representative estimates. These exclusions led to a final analytic sample of 7409 participants.

### Ethical Considerations

This is a secondary analysis of data collected by the ABCD Study. The ABCD Study was approved by the central Institutional Review Board (IRB) of the University of California, San Diego (IRB# 160091) and by the IRB at each of the 21 participating research sites [59,60]. Written informed consent was obtained from all parents or legal guardians prior to data collection [59,60]. This analysis used the deidentified, publicly available ABCD Study dataset obtained through the NIH Data Hub, and it was deemed exempt from human subject review by the investigators’ IRB (Indiana University Bloomington; 2008226356). Participants and families were compensated for the time spent participating in the study, with amounts varying by data collection site.

### Measurements

#### Outcome Variables

##### Positive Expectancies of Cannabis Use Effects

Positive expectancies of cannabis use effects were assessed using youth self-report on the Marijuana Effect Expectancy Questionnaire-Brief (MEEQ-B) [25]. The items assessed the degree to which adolescents believe that (1) “marijuana helps a person relax and feel less tense,” (2) “marijuana helps people get along better with others or feel more romantic,” and (3) “marijuana enhances creativity or alters perceptions.” The MEEQ-B has been validated among adolescents and young adults, effectively capturing beliefs about the effects of cannabis [61]. Youth responded to 3 positive expectancy questions on a 5-point Likert scale, with higher summed scores (range 3‐15) reflecting stronger positive expectancies. Internal consistency (Cronbach α) for the positive expectancies of cannabis use effects scale indicated good reliability (α=.77 [T1], .80 [T2], and .83 [T3]).

### Predictor Variables

#### Family Cannabis Use Rules

Aligning with previous studies [62-64], cannabis use rules were measured using parental report on the following question: “What are the family rules about using marijuana for your son/daughter?” [65-68] Responses were dichotomized as “strict rules” (“not allowed to use marijuana under any circumstances”) versus “lenient/no rules,” which included all other responses (ie, “not allowed to use marijuana in the home but no rules outside the home,” “allowed to use marijuana in the home with permission,” “allowed to use marijuana in the home whenever desired,” “no rules set about marijuana use,” and “have not yet made rules about my child’s marijuana use”). Given our study’s focus on cannabis and the high correlation of family rules regarding cannabis, alcohol, and nicotine use (*r*>0.70), alcohol- and nicotine-specific rules were excluded from the present analyses to minimize multicollinearity and improve model interpretability.

#### Parental Monitoring

Parental monitoring was assessed using youth self-report on 4 items measured on a 5-point Likert scale (1=Never, 2=Almost never, 3=Sometimes, 4=Often, and 5=Always or almost always), with higher mean scores (range 1‐5) indicating greater parental knowledge, involvement, oversight, and communication [69]. The four items were as follows: (1) “How often do your parents/guardians know where you are?” (2) “If you are at home while your parents or guardians are away, how often do you know how to contact them?” (3) “How often do you talk to your parents or guardians about your plans for the following day, such as school activities or other engagements?” and (4) “How many times do you and your parents/guardians eat dinner together?” This measure reflects the widely used conceptualization of parental monitoring in adolescent development research [40,70-72].

#### Family Conflict

Consistent with previous studies using the ABCD Study dataset for developmental research [47,72-74], family conflict was assessed using youth self-report on 9 items from the Family Conflict Subscale of the ABCD Study Parent Family Environment Scale, adapted from the PhenX toolkit [75]. Items were coded as True=1 and False=0, with reverse coding applied to positively worded items. The following items were included: “We fight a lot in our family” (1=True), “Family members sometimes get so angry they throw things” (1=True), “Family members often criticize each other” (1=True), and “Family members sometimes hit each other” (1=True). Reverse-coded items included statements such as “Family members rarely become openly angry” (1=False), “Family members hardly ever lose their tempers” (1=False), “If there’s a disagreement in our family, we try hard to smooth things over and keep the peace” (1=False), and “In our family, we believe you don’t ever get anywhere by raising your voice” (1=False). The items were averaged together, with higher mean scores indicating greater conflict (range 0‐9). Internal consistency values (Cronbach α) for this study were .67 (T1), .64 (T2), and .68 (T3).

### Covariates

#### Psychopathology

Consistent with previous studies [76-79], youth psychopathology was assessed with parent-reported standardized total scores from the Child Behavior Checklist (CBCL) [80]. This questionnaire comprises 112 items rated on a 3-point Likert scale (0=Not at all true, 1=Somewhat true, and 2=Very true). The total *t* scores were adjusted for age and sex norms derived from population studies, ensuring comparability across participants [79]. Higher *t* scores reflect more severe psychopathological problems (range 24‐88). Cronbach α was .95 for each time point.

#### Demographic Covariates

Demographic covariates included participant age (in years), biological sex assigned at birth (male/female), parent-reported race and ethnicity, and parental highest education and household income [40,55,73,74,81]. Following the ABCD Study–provided race-ethnicity variable and established frameworks developed by sociocultural literature using the ABCD Study [73,82], parent-reported youth race and ethnicity were categorized as Hispanic, non-Hispanic White, non-Hispanic Black, non-Hispanic Asian, and non-Hispanic other/mixed race (including youth whose parents selected multiple racial categories or “Other race”) [83]. Parental education was dichotomized as high school or less versus some college or higher, and household income was dichotomized as less than US $75,000 versus US $75,000 or higher [84-86].

#### State Recreational Cannabis Legalization Status

State recreational cannabis legalization status was coded as legal (Yes) or not legal (No) by the ABCD Study administration based on the participant’s state of residence at baseline in the ABCD Study (approximately 1 year before study T1). Because the dataset does not include time-varying recreational cannabis use policy indicators, this baseline measure served as a proxy for legalization status at T1 (study reference time point).

### Statistical Analysis

For the descriptive analysis, unweighted frequencies and weighted proportions were assessed for categorical variables, and weighted means with SDs were calculated for continuous variables at each time point. Differences across time points were evaluated using weighted chi-square tests for categorical variables and weighted ANOVA for continuous variables. Prior to modeling the latent growth models, a bivariate correlation matrix was examined to assess multicollinearity between predictors.

A series of latent growth models was fitted to examine developmental trajectories of positive expectancies of cannabis use effects. Unconditional latent growth curve models (LGCMs) were first examined to assess within-person change and determine whether sufficient heterogeneity existed to justify latent class modeling [50,87,88]. Both linear and quadratic LGCMs were tested using maximum likelihood estimation with robust SEs.

Subsequently, LCGA models were used to identify distinct subgroups of adolescents with similar trajectories of positive expectancies of cannabis use effects. Consistent with standard practice, all the LCGA models were specified with intercept and slope variances fixed to zero and residual variances constrained to equality across time points [54]. Unconditional LCGA models with 1 to 7 classes were evaluated to assess the optimal number of trajectory classes. Model fit was assessed using multiple criteria: Akaike information criterion (AIC), Bayesian information criterion (BIC), sample size–adjusted Bayesian information criterion (aBIC), entropy, and Lo-Mendell-Rubin adjusted likelihood ratio test (LMR-aLRT) [89-91]. The optimal model was defined as having the lowest information criterion values, significant LMR-aLRT, entropy ≥0.80, and no class size smaller than 5% of the total sample, which was considered statistically unstable [52].

After determining the best-fitting model, R3STEP (auxiliary procedure that implements the 3-step method for adding predictors of latent class membership specified by Mplus) was applied to examine associations between class membership and time-invariant covariates (ie, age, biological sex, race/ethnicity, parental education, recreational cannabis legal status, and total family income). This procedure accounts for the uncertainty in class assignments by incorporating posterior probabilities into auxiliary multinomial logistic regressions. This approach improves estimation accuracy and protects against biased parameter estimates [92,93]. Race/ethnicity was specified as a nominal variable in Mplus, which dummy-coded the variable using non-Hispanic White as the reference category. Mplus then reported the overall omnibus effect of the race/ethnicity block rather than separate coefficients for each category unless individual dummy-coded contrasts produced statistically separable estimates across class comparisons. The full set of dummy contrasts nevertheless contributed internally to the estimation of the classification error–adjusted multinomial logistic model.

To further explore predictive associations with time-varying variables (ie, family cannabis rules, parental monitoring, family conflict, and psychopathology), 2 complementary models were estimated. A class-specific effects model was used to assess the different effects of time-varying predictors across latent classes without interfering with the predefined trajectory classes. This approach revealed heterogeneity in the associations between time-varying predictors and outcome variables across developmental trajectories. As a sensitivity analysis, a common effects model was used to estimate population-average associations between the time-varying predictors and outcome variables under the assumption that the effects of the time-varying predictors on the outcome variables are homogeneous across all latent classes. This model provides insights to understand the general exposure effects that are consistent across subpopulations.

All LCGA models were estimated using Mplus 8.11 [94]. To ensure model stability and reduce the risk of convergence on the local maxima, a multistage estimation procedure was used. Each model was initialized with 1000 random sets of starting values, from which the 250 best-fitting solutions were retained for final optimization. To further verify solution stability, log-likelihood values were required to replicate across 20 iterations. Likelihood ratio tests (eg, LMR-aLRT) were conducted with an additional set of 1000 random start replications, with 200 used for preliminary evaluation and 500 selected for final optimization, repeated 100 times, to ensure the reliability of model comparison results. Missing data of predictors were imputed using the nonparametric random forest–based approach, which has been shown to perform well in retaining nonlinear relationships and interactions among variables in mixed-type datasets [95]. Descriptive statistics were conducted with R 4.5.0 (via RStudio, Posit). The reporting of this study followed the STROBE (Strengthening the Reporting of Observational Studies in Epidemiology) guidelines (Checklist 1) [96].

## Results

### Descriptive Statistics

Table 1 presents the descriptive statistics of the study sample across the 3 time points. The positive expectancies of cannabis use effects score measured among the participants demonstrated an increasing trend (T1: mean 6.41, SD 2.85; T2: mean 7.20, SD 2.93; T3: mean 7.96, SD 2.99; *P*<.001). Similarly, the proportion of strict family cannabis use rules increased over time from 97.9% (T1) to 98.9% (T3) (*P*<.001). Moreover, family conflict scores increased from 1.90 (SD 1.88) to 2.09 (SD 1.96) (*P*<.001). Parental monitoring scores showed a slight decrease across waves (T1: mean 4.51, SD 0.45; T3: mean 4.38, SD 0.50; *P*<.001).

**Table 1.** Descriptive statistics of participants from time 1 to time 3 in the Adolescent Brain Cognitive Development Study (ABCD Study) (N=7409).

Variable^a^	Time 1 (age 10-11 years)^b^	Time 2 (age 11-12 years)^b^	Time 3 (age 12-13 years)^b^	*P* value^c^
Positive expectancies of cannabis use effects (range 3-15), mean (SD)	6.41 (2.85)	7.20 (2.93)	7.96 (2.99)	<.001
Biological sex, n (%)				—
Male	4040 (49.0)	—^d^	—	
Female	3369 (51.0)	—	—	
Race/ethnicity, n (%)				—
NH^e^ White	4126 (20.7)	—	—	
NH Black	918 (37.6)	—	—	
Hispanic	1421 (28.9)	—	—	
NH Asian	144 (0.4)	—	—	
NH others	800 (12.3)	—	—	
Parental education, n (%)				—
High school or less	1163 (21.5)	—	—	
Some college or higher	6221 (78.5)	—	—	
Recreational cannabis legal status^f^, n (%)				—
No	5114 (72.8)	—	—	
Yes	1973 (27.2)	—	—	
Total family income, n (%)				—
Less than US $75,000	2647 (74.4)	—	—	
US $75,000 or higher	4269 (25.6)	—	—	
Age (range 7‐16 years), mean (SD)	10.55 (0.64)	11.56 (0.71)	12.51 (0.68)	<.001
Standardized psychopathology *t* score (range 24-88), mean (SD)	45.67 (11.06)	45.11 (11.13)	45.01 (11.25)	<.001
Family conflict score (range 0‐9), mean (SD)	1.90 (1.88)	1.90 (1.84)	2.09 (1.96)	<.001
Parental monitoring score (range 1‐5), mean (SD)	4.51 (0.45)	4.50 (0.46)	4.38 (0.50)	<.001
Family cannabis use rules, n (%)				<.001
Lenient/no rules	1685 (2.3)	1331 (1.8)	1010 (1.1)	
Strict rules	5712 (97.9)	6033 (98.2)	6207 (98.9)	

a Except for the baseline sociodemographic characteristics (biological sex, race/ethnicity, parental education, and recreational cannabis legal status), all other variables were measured repeatedly from time 1 (T1) to time 3 (T3).

b Values represent unweighted frequencies and weighted proportions for categorical variables, and weighted means with SDs for continuous variables. Frequencies may not sum to the total sample size due to missing data.

c *P* values were generated using weighted ANOVA for continuous variables and weighted chi-square tests for categorical variables to test differences across waves.

d Not applicable.

e NH: non-Hispanic.

f The cannabis recreational legal status was determined based on the participant’s state of residence at the time of their baseline interview in the ABCD Study, which is approximately 1 year prior to T1 in this study.

### Unconditional LCGA Model Statistics Regarding Positive Expectancies of Cannabis Use Effects

Table 2 presents the latent class model fit comparisons of the optimal class solutions, ranging from 1 class to 7 classes. Table 3 presents the sizes of the individual classes. While models with a greater number of classes (5-class to 7-class trajectory solutions) were explored, they yielded subgroups with minimal representation (ie, group size <5% of the total sample), raising concerns about model overfitting and limited interpretability.

**Table 2.** Latent class model fit comparisons for unconditional latent class growth analysis models regarding positive expectancies of cannabis use effects.

Trajectory (model)	Log likelihood	BIC^a^	aBIC^b^	AIC^c^	LMR-aLRT^d^ *P* value	BLRT^e^ *P* value	Entropy^f^	Minimal class membership^g^ (%)
1 class	−55350.179	110744.910	110729.021	110710.358	—^h^	—	—	—
2 classes	−53156.244	106383.722	106358.349	106328.488	<.001	<.001	0.734	38.9
3 classes	−52609.740	105317.495	105282.540	105241.480	<.001	<.001	0.776	16.9
4 classes^i^	−52116.646	104358.038	104313.549	104261.292	<.001	<.001	0.827	9.2
5 classes	−51746.873	103645.224	103591.202	103527.746	<.001	<.001	0.841	4.4
6 classes	−51583.031	103344.270	103280.714	103206.061	<.001	<.001	0.838	2.9
7 classes	−51396.675	102998.291	102925.202	102839.351	<.001	<.001	0.866	0.4

a BIC: Bayesian information criterion.

b aBIC: sample size–adjusted Bayesian information criterion.

c AIC: Akaike information criterion.

d LMR-aLRT: Lo-Mendell-Rubin adjusted likelihood ratio test; *P* value for k-1 refers to a significant improvement in model fit between the class (k) and the preceding class (k-1), which compares whether a profile solution with k profiles fits significantly better than a profile.

e BLRT: parametric bootstrapped likelihood ratio test, which is similar to the LMR-aLRT; *P* value refers to a significant improvement in model fit between the class (k) and the preceding class (k-1).

f Entropy indicates classification accuracy, with a higher value indicating better classification (range 0-1).

g Minimal class membership represents the proportion of participants in the latent class with the smallest membership.

h Not applicable.

i Selected model.

**Table 3.** Sizes of the classes (N=7409).

Trajectory (model)	Class^a^						
	Class 1, n (%)	Class 2, n (%)	Class 3, n (%)	Class 4, n (%)	Class 5, n (%)	Class 6, n (%)	Class 7, n (%)
1 class	7409 (100.0)	—^b^	—	—	—	—	—
2 classes	4530 (61.1)	2879 (38.9)	—	—	—	—	—
3 classes	4125 (55.7)	2035 (27.5)	1249 (16.9)	—	—	—	—
4 classes^c^	2111 (28.5)	684 (9.2)	1496 (20.2)	3118 (42.1)	—	—	—
5 classes	1897 (25.6)	328 (4.4)	3054 (41.2)	1470 (19.8)	660 (8.9)	—	—
6 classes	1864 (25.2)	674 (9.1)	502 (6.8)	1375 (18.6)	2774 (37.4)	220 (2.9)	—
7 classes	1247 (16.8)	31 (0.4)	1439 (19.4)	219 (2.9)	1433 (19.3)	2398 (32.4)	642 (8.7)

a Each cell displays the frequency and corresponding proportion of individuals within each latent class. Frequencies represent the unweighted counts, while proportions are calculated relative to the total number within each class or group.

b Not applicable.

c Selected model.

The 4-class model was selected as the optimal solution based on both statistical fitness and conceptual interpretability. This model demonstrated comparatively lower values for the BIC, aBIC, and AIC and higher entropy compared with the 3-class model, indicating improved classification precision. Compared with the 5-class model, it maintained a balanced class distribution, with each subgroup exceeding the recommended 5% minimum threshold (the smallest group being class 2, with 684 cases or 9.2% of the total sample). Therefore, the 4-class solution balanced parsimony with meaningful subgroup differentiation, which avoided interpretive challenges posed by extremely small latent classes observed in higher-order models.

**Figure 1.**
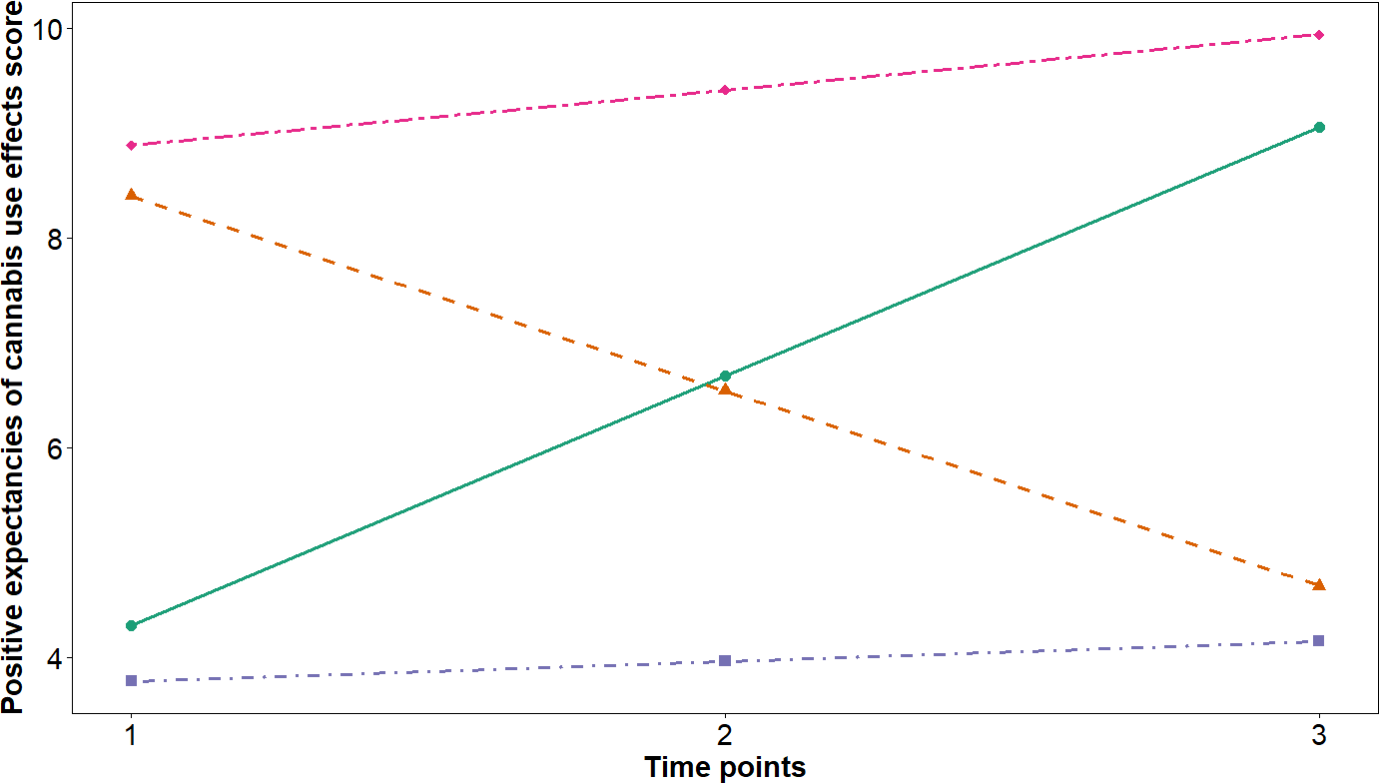
The 4-class developmental trajectories of positive expectancies of cannabis use effects at 3 time points. The y-axis represents the mean positive expectancies of cannabis use effects score, and the x-axis represents the 3 examined time points. The 4 trajectories represent latent classes identified through latent class growth modeling: class 1 is plotted with a solid green line and filled circle markers (2111/7409, 28.5%), class 2 is plotted with an orange dashed line and filled triangle markers (684/7409, 9.2%), class 3 is plotted with a purple dot-dash line and filled square markers (1496/7409, 20.2%), and class 4 is plotted with a pink 2-dash line and filled diamond markers (3118/7409, 42.1%).

Figure 1 visualizes the 4-class trajectories. The largest subgroup followed a moderate-increasing trajectory that was characterized by a high baseline level but a moderate increasing trend over time (class 4: moderate-increasing class; n=3118, 42.1% of the sample). The second most prevalent trajectory followed a high-increasing trajectory that was characterized by a moderate baseline level and a steep increase across the study period (class 1: high-increasing class; n=2111, 28.5% of the sample). The third most prevalent trajectory followed a low-increasing trajectory that was characterized by a low baseline level with a slight increase (class 3: low-increasing class; n=1496, 20.2% of the sample). The smallest group followed a high-decreasing trajectory that was characterized by a high baseline level that declined sharply (class 2: high-decreasing class; n=684, 9.2% of the sample). Parameter estimates and detailed trajectory features are reported in Table S3 in Multimedia Appendix 1.

### Associations Between Baseline Time Invariant Variables and Latent Class Membership

Table 4 presents the results of multinomial logistic regression with the low-increasing class (class 3) serving as the reference category. Compared with this group, youth in the high- and moderate-increasing classes were older (adjusted odds ratio [aOR] 1.13, 95% CI 1.01‐1.25; *P*=.04 and aOR 1.45, 95% CI 1.31‐1.60; *P*<.001, respectively). Those in the moderate-increasing class were also more likely to reside in states with legalized recreational cannabis use and be from higher-income families. Multinomial logistic regression findings with other groups as reference categories are reported in Tables S5‐S7 in Multimedia Appendix 1.

**Table 4.** Multinomial logistic regression predicting latent class membership (reference class: class 3).

Class^a^ and variable^b^		aOR^c^^,^^d^ (95% CI)		*P* value
Class 1				
Biological sex		0.94 (0.82‐1.08)		.39
Race/ethnicity		1.03 (0.98‐1.08)		.27
Parental education		1.06 (0.88‐1.28)		.53
Recreational cannabis legal status		1.13 (0.96‐1.32)		.16
Age		1.13 (1.01‐1.25)		.04^e^
Total family income		1.00 (0.87‐1.16)		.97
Class 2				
Biological sex		1.05 (0.88‐1.27)		.59
Race/ethnicity		0.97 (0.90‐1.04)		.40
Parental education		0.85 (0.67‐1.09)		.17
Recreational cannabis legal status		1.06 (0.85‐1.32)		.60
Age		0.99 (0.85‐1.14)		.88
Total family income		1.09 (0.90‐1.32)		.40
Class 4				
Biological sex		1.01 (0.89‐1.15)		.84
Race/ethnicity		1.02 (0.97‐1.07)		.38
Parental education		0.99 (0.83‐1.17)		.88
Recreational cannabis legal status		1.28 (1.11‐1.49)		.003^f^
Age		1.45 (1.31‐1.60)		<.001^g^
Total family income		1.25 (1.09‐1.42)		.004^f^

a The reference category for this model is class 3 (low-increasing), which represents 1496 participants (20.2%).

b For covariates, the reference groups are as follows: female for biological sex, non-Hispanic White for race/ethnicity, high school education or less for parental education, non-legalized status for recreational cannabis legal status, and total family income below US $75,000 for family income. Race/ethnicity was specified as a 5-category nominal covariate and was dummy-coded internally by Mplus. Mplus reports a single omnibus effect representing the overall effect of this multicategory covariate.

c aOR: adjusted odds ratio.

d Reported odds ratios represent the relative odds of belonging to each latent class versus the reference class for each covariate category.

e *P*<.05.

f *P*<.01.

g *P*<.001.

### Dynamic Associations of Family Environment and Psychopathology With the Trajectories of Positive Expectancies of Cannabis Use Effects Within Each Latent Trajectory Class

Table 5 presents the results from the class-specific effects model estimating the time-varying associations of familial and psychopathological predictors with positive expectancies of cannabis use effects across the 4 identified latent trajectory classes. Each class was modeled independently to capture heterogeneity in relation to the predictors over time. Distinct time-varying familial and psychopathological predictors emerged, underscoring differential developmental processes. In the high-increasing class, lower parental monitoring predicted greater expectancy growth at both T1 (β=−0.152, SE=0.072; *P*=.04) and T2 (β=−0.477, SE=0.122; *P*<.001), while increased family conflict at T3 (β=0.071, SE=0.019; *P*<.001) predicted elevated positive expectancies of cannabis use effects. In the high-decreasing class, only family conflict at T2 (β=0.124, SE=0.063; *P*=.047) was a significant risk factor, possibly reflecting transient reinforcement of positive expectancies before decline. The low-increasing class exhibited no significant associations across time points, though family conflict at T3 approached significance (β=0.038, SE=0.021; *P*=.07). In contrast, the moderate-increasing class (the largest group) showed the most consistent effects: less strict family cannabis use rules were significantly associated with expectancy increases at T1 (β=−0.171, SE=0.063; *P*=.006) and T2 (β=−0.212, SE=0.091; *P*=.02), lower parental monitoring was significant at both T2 (β=−0.275, SE=0.088; *P*=.002) and T3 (β=−0.307, SE=0.066; *P*<.001), and family conflict was the most robust predictor from T1 to T3 (*P*<.005).

**Table 5.** Class-specific estimates of time-varying predictors of positive expectancies of cannabis use effects across 3 time points.

Class, time, and variable^a^			β	SE	z-statistics	*P* value
Class 1 (high-increasing trajectory)						
Time 1						
Family cannabis use rules			−0.079	0.071	−1.108	.27
Parental monitoring			−0.152	0.072	−2.111	.04^b^
Psychopathology *t* score			−0.004	0.003	−1.293	.20
Family conflict			−0.016	0.017	−0.959	.34
Time 2						
Family cannabis use rules			−0.226	0.149	−1.519	.13
Parental monitoring			−0.477	0.122	−3.922	<.001^c^
Psychopathology *t* score			−0.008	0.006	−1.312	.19
Family conflict			0.019	0.033	0.587	.56
Time 3						
Family cannabis use rules			0.023	0.099	0.229	.82
Parental monitoring			−0.123	0.077	−1.603	.11
Psychopathology *t* score			0.005	0.004	1.527	.13
Family conflict			0.071	0.019	3.687	<.001^c^
Class 2 (high-decreasing trajectory)						
Time 1						
Family cannabis use rules			−0.143	0.141	−1.009	.31
Parental monitoring			0.048	0.139	0.349	.73
Psychopathology *t* score			−0.004	0.006	−0.765	.44
Family conflict			−0.007	0.037	−0.195	.85
Time 2						
Family cannabis use rules			0.150	0.275	0.544	.59
Parental monitoring			−0.369	0.267	−1.381	.17
Psychopathology *t* score			−0.006	0.010	−0.620	.54
Family conflict			0.124	0.063	1.982	.047^b^
Time 3						
Family cannabis use rules			−0.123	0.169	−0.729	.47
Parental monitoring			−0.135	0.129	−1.044	.30
Psychopathology *t* score			−0.010	0.006	−1.825	.07
Family conflict			0.050	0.033	1.500	.13
Class 3 (low-increasing trajectory)						
Time 1						
Family cannabis use rules			−0.065	0.073	−0.886	.38
Parental monitoring			−0.063	0.064	−0.978	.33
Psychopathology *t* score			0.004	0.003	1.408	.16
Family conflict			0.013	0.017	0.801	.42
Time 2						
Family cannabis use rules			0.099	0.150	0.661	.51
Parental monitoring			−0.143	0.119	−1.204	.23
Psychopathology *t* score			0.002	0.005	0.410	.68
Family conflict			0.040	0.036	1.111	.27
Time 3						
Family cannabis use rules			0.074	0.107	0.697	.49
Parental monitoring			−0.045	0.069	−0.645	.52
Psychopathology *t* score			−0.002	0.003	−0.814	.42
Family conflict			0.038	0.021	1.805	.07
Class 4 (moderate-increasing trajectory)						
Time 1						
Family cannabis use rules			−0.171	0.063	−2.733	.006^d^
Parental monitoring			−0.089	0.068	−1.317	.19
Psychopathology *t* score			0.004	0.003	1.555	.12
Family conflict			0.046	0.016	2.859	.004^d^
Time 2						
Family cannabis use rules			−0.212	0.091	−2.317	.02^b^
Parental monitoring			−0.275	0.088	−3.105	.002^d^
Psychopathology *t* score			0.012	0.004	2.908	.004^d^
Family conflict			0.079	0.022	3.689	<.001^c^
Time 3						
Family cannabis use rules			0.031	0.078	0.402	.69
Parental monitoring			−0.307	0.066	−4.654	<.001^c^
Psychopathology *t* score			0.010	0.003	3.085	.002^d^
Family conflict			0.074	0.016	4.661	<.001^c^

a The outcome variable is the individual’s level of positive expectancies of cannabis use effects at each time point (time 1, time 2, and time 3) within each latent class. All covariates are repeated measures within respondents from time 1 to time 3. This model does not predict the growth trajectory, but instead, it estimates how time-varying predictors are associated with variation in the positive expectancies of cannabis use effects score over time within each trajectory class.

b *P*<.05.

c *P*<.001.

d *P*<.01.

### Shared Associations of Family Environment and Psychopathology With Positive Expectancies of Cannabis Use Effects Across Latent Trajectory Classes

Results from the common effects model are presented in Table S8 in Multimedia Appendix 1, where time-varying familial and psychopathological predictors were constrained to have equal influence across all latent trajectory classes. Strict family cannabis use rules were significantly associated with lower positive expectancies of cannabis use effects at T1 only (β=−0.130, SE=0.040; *P*=.001). However, parental monitoring remained a significant predictor for positive expectancies of cannabis use effects across all 3 time points (T1: β=−0.089, SE=0.040; *P*=.03; T2: β=−0.315, SE=0.061; *P*<.001; T3*:* β=−0.185, SE=0.040; *P*<.001). Family conflict was a consistent and robust risk factor, with its influence increasing from T2 to T3 (*P*<.001).

## Discussion

### Heterogeneous Trajectories of Positive Expectancies of Cannabis Use Effects in Early Adolescence

To the best of our knowledge, this is the first study to use a large-scale longitudinal dataset to examine the developmental trajectories of positive expectancies of cannabis use effects in early adolescents, using a person-centered analytic framework. By modeling the development of positive expectancies of cannabis use effects across early adolescence, this study offers novel insights into the dynamic, heterogeneous nature of the formation of positive expectancies of cannabis use effects during this sensitive developmental phase. It identified the following 4 distinct trajectories of positive expectancies of cannabis use effects: high-increasing, high-decreasing, low-increasing, and moderate-increasing trajectories. These trajectories highlight the substantial variability in both the baseline levels and patterns of change in positive expectancies of cannabis use effects, underscoring early adolescence as an important period for tailoring interventions to prevent cannabis use.

Although weighted descriptive statistics across the sample indicated relatively modest population-level changes over the 3 waves, this pattern is expected given the narrow but critical developmental window of early adolescence (approximately ages 11‐13 years) represented in our sample from the ABCD Study cohort. During this period, many psychosocial and contextual characteristics exhibit relative stability at the population level [97], yet substantial *within-person* variability persists in cognitive-affective processes such as substance-related expectancies, social-emotional development, and dynamic familial factors [7,98-100]. LCGA, a person-centered approach, is uniquely suited to capturing individual-level heterogeneity because it identifies subgroups of youth who share similar developmental trajectories even when the overall mean trend appears relatively flat. Accordingly, the 4 trajectory classes identified in this study represent distinct and meaningful expectancy development over time rather than simple cross-sectional differences based on average levels [52,101]. In addition, although the trajectory classes are derived from repeated measures of positive expectancies of cannabis use effects, the LCGA analytic framework used in this study helps minimize potential bias arising from factors, such as parental monitoring and family conflict, which may influence both the development of positive expectancies of cannabis use effects and the predictors included in the R3STEP model. LCGA does not stratify individuals on a single observed positive expectancies of cannabis use effects score; instead, it forms subgroups based on model-estimated posterior probabilities that reflect the overall pattern of trajectories. Moreover, R3STEP estimates covariate associations only after class formation and incorporates adjustment for classification uncertainty, reducing the bias that can arise when treating uncertain class assignments as if they are certain [92]. Within this framework, associations between predictors and class membership represent correlational patterns among latent developmental pathways rather than artifacts of the analytic strategy. Thus, the heterogeneity observed across classes reflects meaningful differences in expectancy development over time, highlighting the advantage of mixture modeling for uncovering nuanced developmental processes that would remain obscure in traditional variable-centered analyses.

Using this approach, the largest trajectory class identified in this study was the moderate-increasing trajectory, which was characterized by relatively high initial levels of positive expectancies of cannabis use effects that increased steadily with time, suggesting an active expectancy formation phase. This pattern may reflect normative developmental processes in early adolescence, where adolescents increasingly seek self-identity and autonomy, and become increasingly susceptible to substance use opportunities [102]. Multivariable comparisons revealed that youth in this trajectory were more likely to be older, from high-income families, and living in states with recreational cannabis legalization. Notably, the consistently elevated and gradually intensifying positive expectancies of cannabis use effects observed in this group are concerning, as the findings point to a sustained expectancy formation process that may heighten the risk for future initiation and persistent use. Youth in this trajectory may be actively shaping their cognitive belief around cannabis use prior to engaging in cannabis use, which may be reinforced by their developmental maturity (older age) and the legal status of recreational cannabis use in their environment. These findings suggest that prevention efforts should extend beyond traditionally high-risk youth to include those on seemingly normative developmental pathways who may nonetheless be building pro-cannabis expectancies that increase long-term vulnerability. The other 3 trajectories provide further insights into heterogeneity in the development of positive expectancies of cannabis use effects, highlighting the substantial variability in both the onset and developmental course of positive expectancies of cannabis use effects and pointing to multiple pathways of risk and resilience in early cannabis-related cognitions.

### Familial Protective and Risk Factors

Findings from the common effects model showed that stricter family cannabis use rules and higher levels of parental monitoring were protective against positive expectancies of cannabis use effects during early adolescence. These effects were the strongest at earlier ages, particularly at 10‐11 years (T1) and 11‐12 years (T2). Parental monitoring demonstrated a consistent inverse association with positive expectancies of cannabis use effects across all 3 time points, with the strongest effect at ages 11‐12 years. In contrast, cannabis-specific rules were significant only at T1, with diminished predictive value at later time points. These protective effects align with the findings of a large body of developmental research emphasizing the critical role of structured and engaged parenting in deterring adolescent substance-related cognitions and behaviors [43,103].

The early and pronounced influence of family cannabis use rules underscores their importance in shaping adolescents’ cognitive attitudes regarding substance use when they are most embedded within the family context and are more receptive to parental expectations and boundaries [104]. These rules function as clear behavioral norms, potentially counterbalancing early exposure to peer influences and emerging social scripts around cannabis. Although the direct statistical effect weakened by ages 11‐12 years, such rules may establish enduring internalized norms that persist even when external risks are present. Prior research suggests that early parental rule-setting exerts long-term influence on substance-related decision-making. For example, the authoritative parenting style is characterized by setting limits and is linked to lower substance use and less positive attitudes toward drugs throughout adolescence [105-107].

Parental monitoring demonstrated a more enduring and stable protective effect across the developmental period studied. Unlike rule-setting, monitoring reflects an ongoing dynamic engagement with the daily lives of adolescents, which provides not only behavioral oversight but also emotional attunement and accountability [108]. This form of proactive parenting has been consistently shown to reduce adolescents’ opportunities to engage in risk behaviors and to shape substance-related cognitions in a protective direction [65,108]. The heightened impact observed at ages 11-12 years may indicate a critical developmental “sweet spot,” when adolescents begin to seek autonomy but remain highly responsive to external regulation and support. These findings emphasize the importance of initiating family-based prevention efforts during early adolescence, leveraging this window to reinforce cognitive resistance to substance use before peer norms and societal influences exert stronger effects.

Family conflict emerged as a robust risk factor for elevated positive expectancies of cannabis use effects at ages 12-13 years (T3). In addition, class-specific models showed that this effect was the strongest in the moderate- and high-increasing classes, suggesting that sustained family tensions may accelerate the formation of positive expectancies of cannabis use effects, particularly when not offset by protective parenting practices. This finding is consistent with the findings of a substantial body of literature linking family dysfunction to increased vulnerability to substance use [109,110]. Chronic conflict may erode emotional regulation capacities and model maladaptive coping strategies, thereby reinforcing cannabis as a perceived tool for managing stress [111]. These findings highlight the dual importance of reinforcing protective parenting practices and reducing family conflict during this critical developmental period.

### Class-Specific Nuances

Unlike the common effects model, which assumes that predictors operate uniformly across all trajectories, the class-specific model allows the effects of parental and familial factors to vary by trajectory and captures heterogeneity in developmental processes. The findings revealed notable differences in the influences of familial and psychopathological factors across the 4 trajectories of positive expectancies of cannabis use effects.

Parental monitoring was pronounced among adolescents who followed trajectories marked by moderate- and high-increasing risk, where higher monitoring during early and mid-adolescence (ages 10‐12 years) was associated with significantly lower positive expectancies of cannabis use effects. Additionally, stricter family cannabis use rules were associated with lower positive expectancies of cannabis use effects in the moderate-increasing group at ages 10-11 and 11-12 years. These effects were most pronounced prior to the age of 13 years, underscoring a sensitive developmental window when parental guidance may shape substance-related cognitions before peer norms and autonomy-seeking dominate [112,113]. It is also possible that elevated parental monitoring reflects a reactive process, wherein parents increase oversight in response to perceiving their child’s heightened risk or early signs of problematic behaviors. In this interpretation, monitoring may serve as a preventive and responsive strategy, suggesting that parents who recognize vulnerability may intensify supervision as a preemptive measure against further risk escalation.

However, these protective effects were less evident or absent in the high-decreasing and low-increasing trajectories. In the high-decreasing group, early high levels of positive expectancies of cannabis use effects declined over time, and neither parental monitoring nor rule-setting was significantly associated with these shifts. This may suggest that reductions in positive expectancies of cannabis use effects were driven by other factors, such as experiential disconfirmation or broader contextual influences, and further research is warranted for this group of youth. In the low-increasing group, neither monitoring nor rule-setting showed significant associations, though family conflict emerged as a marginal risk factor at later ages. The absence of early effects in this group could reflect other protective dispositional factors (eg, low sensation-seeking) or structural buffers (eg, strong school engagement) not captured in our analysis.

Family conflict emerged as the most robust and class-differentiating risk factor across trajectories, particularly for those at elevated or increasing risk. Adolescents in the moderate- and high-increasing groups exhibited significantly elevated positive expectancies of cannabis use effects in association with greater family conflict, especially by ages 12-13 years. This pattern suggests that interpersonal stress within the home may amplify the development of positive expectancies of cannabis use effects during a period of heightened social and emotional reactivity. In line with developmental cascade models [114], chronic exposure to family conflict may erode previously adaptive cognitive trajectories and accelerate the adoption of risk-promoting beliefs. Conflict was also a significant predictor in the high-decreasing group at mid-stage (ages 11-12 years), suggesting a contemporaneous, level-shifting effect of conflict rather than a change in growth rate. In contrast, conflict showed no impact among adolescents in the low-increasing class, potentially reflecting greater resilience or the presence of unmeasured compensatory mechanisms, such as school connectedness and temperamentally based self-regulation.

Psychopathological symptoms played a more nuanced, temporally specific role. Across most trajectories, they were not significant predictors in early adolescence but became increasingly relevant by ages 11-12 and 12-13 years in the moderate-increasing class. This shift may reflect the growing salience of emotional distress in early adolescence, when academic, social, and identity-related demands intensify, and cannabis may be perceived as a coping tool.

Collectively, several effects were close to statistical significance based on the *P* value. It is likely that we detected small effects because of our large sample size. Therefore, the findings warrant replication.

### Limitations

Several limitations should be considered when interpreting the findings of this study. First, the analysis was limited to 3 waves of data collected during early adolescence, which restricts the ability to capture the full developmental trajectory of positive expectancies of cannabis use effects into middle and late adolescence. Given that substance-related attitudes and behaviors often intensify during these later periods, future research with a longer follow-up is needed to determine whether the identified trajectories persist, shift, or predict distal actual cannabis use behavior. Second, the measure of recreational cannabis legalization status was based on state-level policy 1 year prior to this study’s baseline (T1) assessment. While useful as a contextual marker, this static measure may not fully reflect the evolving influence of recreational legalization over time, particularly as policy implementation and social norms continue to change. Third, this study used a 3-step LCGA in which covariates and predictors were deliberately excluded from the trajectory formation process and incorporated only in the R3STEP multinomial logistic regression and subsequent class-specific and common effects models. While this approach preserves the integrity of class estimation, unmeasured or imperfectly measured factors may still be associated with both the trajectory of positive expectancies of cannabis use effects and class membership, introducing the possibility of residual confounding. Fourth, the study was unable to account for a range of other social and contextual influences that are becoming increasingly relevant to adolescent development and substance-related behaviors. Given emerging evidence that cannabis-related content on social media can shape adolescents’ attitudes, expectancies, and perceived norms, future studies should integrate time-matched assessments of digital media exposure to provide a more comprehensive understanding of expectancy formation.

### Public Health Implications

This study fills a critical gap in the literature by identifying 4 distinct developmental trajectories of positive expectancies of cannabis use effects among early adolescents, underscoring the need for prevention strategies that extend beyond universal, one-size-fits-all models. While universal prevention remains important, interventions must be tailored to the heterogeneous developmental pathways identified in this study.

In addition to these practical implications, the observed trajectory patterns and associated family predictors contribute to the development of the current theory. The identification of distinct developmental trajectories of positive expectancies of cannabis use effects suggests that expectancy formation during early adolescence may be heterogeneous rather than uniformly increasing. The findings that stricter household rules and higher parental monitoring were associated with membership in lower or declining classes of positive expectancies of cannabis use effects and that greater family conflict was associated with higher-risk classes of positive expectancies of cannabis use effects are consistent with the social learning theory that emphasizes the role of family environments in shaping evaluative beliefs and related motivational states. Together, these findings suggest that theoretical models of adolescent cannabis use may benefit from incorporating heterogeneity in the development of positive expectancies of cannabis use effects and accounting for the structuring influence of family dynamics during this critical developmental period.

Parental monitoring and clear cannabis use rules were most protective in early adolescence, particularly between the ages of 10 and 12 years, when parental influence remains salient. Intervening during this sensitive period may delay or suppress the rise in positive expectancies of cannabis use effects before peer norms and autonomy-seeking behaviors exert greater influences. These practices are especially critical for adolescents in moderate- and high-increasing trajectories, where sustained parental engagement may disrupt escalation in expectancies. In contrast, family conflict emerged as a robust risk factor, particularly in the later stages of early adolescence. Chronic conflict may amplify cognitive vulnerability, destabilize otherwise adaptive trajectories, and accelerate the internalization of risk-promoting beliefs. Prevention programs that educate parents about conflict management and communication skills could therefore provide meaningful protection. Psychopathology also became increasingly salient by ages 11‐13 years, reinforcing the importance of integrated prevention that includes mental health screening and timely intervention. Addressing emotional distress and teaching adaptive coping strategies may reduce the perceived utility of cannabis for managing stress.

Collectively, these findings highlight the value of early, sustained, and nuanced family involvement across developmental stages. Cannabis use prevention programs should also focus on enhancing parental self-efficacy by providing practical tools for effective communication, conflict resolution, and the implementation of developmentally appropriate cannabis use rules [115]. Early adolescence is a critical period during which parental authority still has a dominant influence, particularly for adolescents not yet embedded in high-risk trajectories. For these adolescents, clear and consistent rule-setting within a supportive context may help prevent escalation in expectancies and delay susceptibility.

## Supplementary material

10.2196/85652 Multimedia Appendix 1 Additional data to support the findings of the study.

10.2196/85652 Checklist 1 STROBE checklist.

## Abbreviations

ABCD Study: Adolescent Brain Cognitive Development Study
aBIC: sample size–adjusted Bayesian information criterion
AIC: Akaike information criterion
aOR: adjusted odds ratio
BIC: Bayesian information criterion
IRB: Institutional Review Board
LCGA: latent class growth analysis
LGCM: latent growth curve model
LMR-aLRT: Lo-Mendell-Rubin adjusted likelihood ratio test
MEEQ-B: Marijuana Effect Expectancy Questionnaire-Brief
NIH: National Institutes of Health
PRIME: plans, responses, impulses, motives, and evaluations
