# Supplementary material for: Developmental Trajectories of Positive Expectancies of Cannabis Use Effects Among Early Adolescents: Longitudinal Observational Study Using Latent Class Growth Analysis
Source: JMIR Public Health Surveill. 2026 Jan 9;12:e85652. doi: 10.2196/85652 (PMC12788717; doi:10.2196/85652)
Supplement: Multimedia Appendix 1 [file publichealth-v12-e85652-s001.docx]

**Multimedia Appendix 1.** Additional data to support the findings of the study.

**Table S1.** Parameter estimates and model fits for unconditional latent growth curve model

|  |  | Estimate | Standard Error | p-value |
| --- | --- | --- | --- | --- |
| Means | Intercept | 6.505 | 0.032 | <0.001^***^ |
|  | Slope | 0.746 | 0.019 | <0.001^***^ |
| Variance | Intercept | 4.126 | 0.164 | <0.001^***^ |
|  | Slope | 1.052 | 0.081 | <0.001^***^ |
| Model Fit Indices | RMSEA | 0.021 |  |  |
|  | CFI | 0.999 |  |  |
|  | TLI | 0.997 |  |  |
|  | SRMR | 0.006 |  |  |

*Notes*: RMSEA = Root Square Error of Approximation, with values <0.05 indicates excellent model fit; CFI = Comparative Fit Index, with values >0.95 are considered as excellent fit; TLI = Tucker-Lewis Index, with values > 0.95 are considered as excellent fit; SRMR = Standardized Root Mean Square Residual, with values <0.05 are considered as excellent fit. Statistical significance (* *p*<0.05, ** *p*<0.01, ****p*<0.001).

As shown in Table S1, an unconditional latent growth curve model was estimated to examine the average trajectory and individual variability in the outcome across the three examined time points prior to conducting a latent class growth analysis. The model demonstrated excellent fit to the data, RMSEA = 0.021 (90% CI: [0.003, 0.043]), CFI = 0.999, TLI = 0.997, and SRMR = 0.006. The estimated mean of the intercept was 6.505 (SE = 0.032, *p* < 0.001), indicating that the average initial level of the outcome at baseline was 6.51. The slope mean was 0.746 (SE = 0.019, *p* < 0.001), suggesting a statistically significant linear increase over time. Significant individual variability was observed in both the intercept (variance = 4.126, *p* < 0.001) and slope (variance = 1.052, *p* < 0.001), indicating heterogeneity in initial status and growth rate across individuals. These findings support the presence of meaningful individual differences in trajectories, warranting further investigation using latent class growth analysis (LCGA) to identify distinct subgroups with similar developmental patterns over time.

**Table S2.** 4-Class average latent class probabilities for most likely latent class membership (row) by latent class (column)

|  | 1 | 2 | 3 | 4 |
| --- | --- | --- | --- | --- |
| 1 | **0.879** | 0.013 | 0.045 | 0.062 |
| 2 | 0.023 | **0.841** | 0.069 | 0.068 |
| 3 | 0.058 | 0.031 | **0.911** | 0.000 |
| 4 | 0.045 | 0.024 | 0.000 | **0.930** |

*Notes:* This table presents the average posterior probabilities for each most likely latent class assignment (rows) by latent class (columns). Diagonal values reflect the average probability of correct classification for participants assigned to a given latent class, indicating classification certainty. Off-diagonal values represent the average probabilities of being assigned to other latent classes, which reflects the extent of misclassification. Higher diagonal values (close to 1.00) suggest strong classification certainty.

Table S2 displays the average latent class probabilities for the most likely latent class membership (rows) by latent class assignment (columns) for the 4-class model. The results indicate a high degree of classification precision, with average probabilities for correctly assigning participants to each class ranging from 0.879 to 0.930 along the diagonal, suggesting strong separation between the identified classes.

**Table S3**. Parameter estimates and features for 4 class trajectories

| Class | Class Identification | N  (%) | Intercept | Slope | *p*-values | Features |
| --- | --- | --- | --- | --- | --- | --- |
| 1 | High increasing | 2111  (28.5%) | 4.30 | 2.38 | *p*<0.001^***^ | Starts moderate and increase rapidly |
| 2 | High decreasing | 684  (9.2%) | 8.40 | -1.86 | *p*<0.001^***^ | Starts high but declines sharply |
| 3 | Low increasing | 1496  (20.2%) | 3.77 | 0.19 | *p*<0.001^***^ | Starts low and increases slowly |
| 4 | Moderate increasing | 3118  (42.1%) | 8.88 | 0.53 | *p*<0.001^***^ | Starts high but increases moderately |

*Notes:*N represents the count of participants for each trajectory and % represents the relative size of each trajectory class within the overall sample*.* The *p*-values demonstrated in the table are for slopes. Statistical significance (* *p*<0.05, ** *p*<0.01, ****p*<0.001).

Table S3 demonstrates the estimated intercepts and slopes for each of the 4 latent trajectory classes of positive expectancies of cannabis use effects over the 3 time points. The high increasing class (Class 1) demonstrated a low starting point (intercept = 4.30) and a significant positive growth trajectory (slope = 2.38, *p*<0.001^***^). The high decreasing class (Class 2) demonstrated a high initial level (intercept = 8.40) but a significant decline over time (slope = -1.86, *p*<0.001^***^). The low stable (Class 3) exhibited a low initial status (intercept = 3.72) with a slight but statistically significant positive slope (0.19, *p*<0.001^***^). Finally, the moderate increase class (Class 4) began at a moderate level (intercept = 8.88) and showed a significant slow increase over time (slope = 0.53, *p*<0.001^***^). These results highlight meaningful heterogeneity in growth trajectories, supporting the identification of distinct developmental patterns across the sample.

**Table S4.** Profile characteristics of early adolescents by cannabis use positive expectancy trajectory class (4-class trajectory model)

|  | Class 1  High increase  (n=2111, 28.5%) | | | Class 2  High decrease  (n=684, 9.2%) | | | Class 3  Low increase  (n=1496, 20.2%) | | | Class 4  Moderate increase  (n=3118, 42.1%) | | |
| --- | --- | --- | --- | --- | --- | --- | --- | --- | --- | --- | --- | --- |
|  | T1 | T2 | T3 | T1 | T2 | T3 | T1 | T2 | T3 | T1 | T2 | T3 |
| Positive expectancies of cannabis use effects (M/SD) | 4.20  (1.32) | 6.95  (2.57) | 9.01  (1.54) | 8.56  (1.54) | 6.14  (2.53) | 4.54  (1.44) | 3.61  (1.06) | 4.42  (2.04) | 3.91  (1.25) | 8.96  (1.54) | 9.16  (2.21) | 10.00  (1.70) |
| Biological Sex |  |  |  |  |  |  |  |  |  |  |  |  |
| Male | 1125  (53.3%) |  |  | 381  (55.7%) |  |  | 818  (54.7%) |  |  | 1716  (55.0%) |  |  |
| Female | 986  (46.7%) |  |  | 303  (44.3%) |  |  | 678  (45.3%) |  |  | 1402  (45.0%) |  |  |
| Race/ethnicity |  |  |  |  |  |  |  |  |  |  |  |  |
| NH-White | 1102  (52.2%) |  |  | 399  (58.3%) |  |  | 804  (53.7%) |  |  | 1821  (58.4%) |  |  |
| NH-Black | 301  (14.3%) |  |  | 86  (12.6%) |  |  | 219  (14.6%) |  |  | 312  (10.0%) |  |  |
| Hispanic | 458  (21.7%) |  |  | 121  (17.7%) |  |  | 306  (20.5%) |  |  | 536  (17.2%) |  |  |
| NH-Asian | 30  (1.4%) |  |  | 11  (1.6%) |  |  | 36  (2.4%) |  |  | 67  (2.1%) |  |  |
| NH-others | 220  (10.4%) |  |  | 67  (9.8%) |  |  | 131  (8.8%) |  |  | 382  (12.3%) |  |  |
| Parental education |  |  |  |  |  |  |  |  |  |  |  |  |
| High school or less | 315  (15.0%) |  |  | 122  (17.9%) |  |  | 235  (15.8%) |  |  | 491  (15.8%) |  |  |
| Some college or higher | 1791  (85.0%) |  |  | 560  (82.1%) |  |  | 1254  (84.2%) |  |  | 2616  (84.2%) |  |  |
| Recreational cannabis legal status |  |  |  |  |  |  |  |  |  |  |  |  |
| No | 1465  (72.7%) |  |  | 482  (74.0%) |  |  | 1073  (75.1%) |  |  | 2094  (70.0%) |  |  |
| Yes | 550  (27.3%) |  |  | 169  (26.0%) |  |  | 356  (24.9%) |  |  | 898  (30.0%) |  |  |
| Age (M/SD) | 10.52  (0.63) | 11.60  (0.71) | 12.51  (0.68) | 10.47  (0.65) | 11.56  (0.69) | 12.45  (0.66) | 10.48  (0.62) | 11.55  (0.69) | 12.45  (0.67) | 10.62  (0.63) | 11.71  (0.71) | 12.62  (0.69) |
| Total family income |  |  |  |  |  |  |  |  |  |  |  |  |
| Less than $75,000 | 805  (40.8%) | 739  (37.6%) | 668  (35.9%) | 238  (37.6%) | 221  (35.4%) | 212  (34.5%) | 562  (40.6%) | 527  (38.3%) | 497  (36.5%) | 1042  (35.6%) | 964  (33.5%) | 897  (32.0%) |
| $75,000 or higher | 1168  (59.2%) | 1224  (62.4%) | 1229  (64.1%) | 395  (62.4%) | 403  (64.6%) | 403  (65.6%) | 822  (59.4%) | 850  (61.7%) | 864  (63.5%) | 1884  (64.4%) | 1910  (66.5%) | 1903  (68.0%) |
| Psychopathology t-score (M/SD) | 45.42  (10.82) | 44.89  (11.00) | 44.87  (11.20) | 45.30  (11.16) | 44.58  (10.95) | 44.77  (11.19) | 44.93  (11.58) | 44.02  (11.69) | 44.02  (11.63) | 45.96  (11.00) | 45.65  (10.75) | 45.66  (11.18) |
| Cannabis use positive expectancy (M/SD) |  |  |  |  |  |  |  |  |  |  |  |  |
| Family conflict (M/SD) | 1.87  (1.88) | 1.93  (1.83) | 2.09  (1.97) | 1.82  (1.77) | 1.82  (1.78) | 1.88  (1.85) | 1.76  (1.79) | 1.62  (1.65) | 1.74  (1.74) | 2.00  (1.92) | 2.06  (1.88) | 2.36  (2.04) |
| Parental monitor (M/SD) | 4.50  (0.45) | 4.47  (0.46) | 4.34  (0.52) | 4.49  (0.43) | 4.52  (0.44) | 4.45  (0.49) | 4.53  (0.46) | 4.54  (0.47) | 4.45  (0.49) | 4.48  (0.43) | 4.46  (0.45) | 4.33  (0.49) |
| Family cannabis use rules |  |  |  |  |  |  |  |  |  |  |  |  |
| Lenient/no rules | 464  (22.0%) | 356  (16.9%) | 263  (12.7%) | 147  (21.5%) | 126  (18.6%) | 89  (13.2%) | 268  (17.9%) | 213  (14.3%) | 150  (10.3%) | 806  (25.9%) | 636  (20.5%) | 508  (16.8%) |
| Strict rules | 1641  (78.0%) | 1746  (83.1%) | 1801  (87.3%) | 537  (78.5%) | 553  (81.4%) | 583  (86.8%) | 1228  (82.1%) | 1275  (85.7%) | 1305  (89.7%) | 2306  (74.1%) | 2459  (79.5%) | 2518  (83.2%) |

*Notes*: This table presents the demographic and behavioral profiles of participants at Time 1 (T1) through Time 3 (T3) across each identified trajectory class. All the variables shown in the table are unweighted repeated measures within study waves from T1 to T3 except for baseline sociodemographic characteristics (age, sex, race/ethnicity). The cannabis recreational legal status was determined based on the participant’s state of residence at the time of their baseline interview at ABCD study, which is approximately one year prior to the T1 of the current study. Psychopathology scale is t-score standardized. NH=non-Hispanic. M= mean, SD = standard deviation. Statistical significance (^***^ *p*< 0.001, ^**^ *p* < 0.01, ^*^ *p* < 0.05).

**Profiles of each latent trajectory class**

Table S4 presents the profile of early adolescents in each of the four latent classes of positive expectancies of cannabis use effects (M/SD) from T1 (age 10/11) to T3 (age 12/13), highlighting the substantial heterogeneity in the developmental risk and socio contextual between identified latent classes. The High Increase class (Class 1, 28.5%) exhibited the lowest initial expectancy scores (M=4.20, SD=1.32), followed by a sharp increase by T3 (M=9.01, SD=1.54). The High Decrease (Class 2; 9.2%) exhibited a notable decline from elevated high initial levels at T1 (M=8.56, SD=1.54) to lower levels at T3 (M=4.54, SD=1.44). The Low Increase class (Class 3; 20.2%) demonstrated low but stable increases over time (T1: M=3.61, SD=1.06; T3: M=3.91, SD=1.25). The Moderate Increasing class (Class 4, 42.1%) demonstrated a modest upward trend (T1: M = 8.96, SD=1.54; T3: M=10.00, SD=1.70).

**Table S5**. Multinomial logistic regression predicting latent class membership (Reference Class: Class 1)

| Class | Variables | aOR | 95% CI | *p*-Value |
| --- | --- | --- | --- | --- |
| C#2 | Biological Sex | 1.16 | [0.94-1.33] | 0.243 |
|  | Race/ethnicity | 0.94 | [0.88-1.01] | 0.081 |
|  | **Parental education** | **0.80** | **[0.64-1.00]** | **0.037^*^** |
|  | Recreational cannabis legal status | 0.94 | [0.77-1.16] | 0.548 |
|  | Age | 0.88 | [0.76-1.01] | 0.053 |
|  | Total family income | 1.09 | [0.91-1.30] | 0.393 |
| C#3 | Biological Sex | 1.06 | [0.93-1.21] | 0.413 |
|  | Race/ethnicity | 0.97 | [0.93-1.02] | 0.253 |
|  | Parental education | 0.94 | [0.78-1.13] | 0.502 |
|  | Recreational cannabis legal status | 0.89 | [0.76-1.04] | 0.111 |
|  | **Age** | **0.89** | **[0.80-0.99]** | **0.019^*^** |
|  | Total family income | 1.00 | [0.87-1.15] | 0.969 |
| C#4 | Biological Sex | 1.07 | [0.96-1.20] | 0.235 |
|  | Race/ethnicity | 0.99 | [0.95-1.04] | 0.746 |
|  | Parental education | 0.93 | [0.79-1.09] | 0.329 |
|  | Recreational cannabis legal status | 1.14 | [1.00-1.29] | 0.068 |
|  | **Age** | **1.28** | **[1.17-1.41]** | **<0.001^***^** |
|  | **Total family income** | **1.24** | **[1.10-1.40]** | **0.001^**^** |

Notes: The reference category for this model is Class 1 (moderate increasing), which represents 2111 participants (28.5%). For predictors, the reference groups are as follows: female for biological sex, non-Hispanic white for race/ethnicity, high school education or less for parental education, non-legalized status for recreational cannabis laws, and total family income below $75,000. Statistical significance (* *p*<0.05, ** *p*<0.01, ****p*<0.001).

**Table S6**. Multinomial logistic regression predicting latent class membership (Reference Class: Class 2)

| Class | Variables | aOR | 95% CI | *p*-Value |
| --- | --- | --- | --- | --- |
| C#1 | Biological Sex | 0.90 | [0.75-1.07] | 0.193 |
|  | Race/ethnicity | 1.06 | [0.99-1.14] | 0.100 |
|  | Parental education | 1.25 | [0.99-1.57] | 0.095 |
|  | Recreational cannabis legal status | 1.06 | [0.87-1.30] | 0.572 |
|  | Age | 1.14 | [0.99-1.31] | 0.089 |
|  | Total family income | 0.92 | [0.77-1.11] | 0.353 |
| C#3 | Biological Sex | 0.95 | [0.79-1.14] | 0.571 |
|  | Race/ethnicity | 1.03 | [0.96-1.11] | 0.410 |
|  | Parental education | 1.17 | [0.92-1.49] | 0.239 |
|  | Recreational cannabis legal status | 0.94 | [0.76-1.17] | 0.576 |
|  | Age | 1001 | [0.87-1.17] | 0.877 |
|  | Total family income | 0.92 | [0.76-1.11] | 0.362 |
| C#4 | Biological Sex | 0.96 | [0.81-1.14] | 0.643 |
|  | Race/ethnicity | 1.05 | [0.99-1.13] | 0.139 |
|  | Parental education | 1.16 | [0.93-1.44] | 0.233 |
|  | Recreational cannabis legal status | 1.21 | [0.99-1.47] | 0.088 |
|  | **Age** | **1.46** | **[1.28-1.68]** | **<0.001^***^** |
|  | Total family income | 1.14 | [0.96-1.36] | 0.170 |

*Notes:* The reference category for this model is Class 2 (high decrease), which represents 684 participants (9.2%). For predictors, the reference groups are as follows: female for biological sex, non-Hispanic white for race/ethnicity, high school education or less for parental education, non-legalized status for recreational cannabis laws, and total family income below $75,000. Statistical significance (* *p*<0.05, ** *p*<0.01, ****p*<0.001).

**Table S7.** Multinomial logistic regression predicting latent class membership (Reference Class: Class 4)

| Class | Variables | aOR | 95% CI | *p*-Value |
| --- | --- | --- | --- | --- |
| C#1 | Biological Sex | 0.93 | [0.83-1.04] | 0.202 |
|  | Race/ethnicity | 1.01 | [0.97-1.05] | 0.748 |
|  | Parental education | 1.08 | [0.92-1.26] | 0.366 |
|  | **Recreational cannabis legal status** | **0.88** | **[0.77-1.00]** | **0.038^*^** |
|  | **Age** | **0.78** | **[0.71-0.85]** | **<0.001^***^** |
|  | **Total family income** | **0.81** | **[0.72-0.91]** | **<0.001^***^** |
| C#2 | Biological Sex | 1.04 | [0.88-1.23] | 0.655 |
|  | Race/ethnicity | 0.95 | [0.89-1.02] | 0.119 |
|  | Parental education | 0.87 | [0.69-1.08] | 0.169 |
|  | **Recreational cannabis legal status** | **0.83** | **[0.68-1.00]** | **0.039^*^** |
|  | **Age** | **0.68** | **[0.60-0.78]** | **<0.001^***^** |
|  | Total family income | 0.88 | [0.73-1.05] | 0.118 |
| C#3 | Biological Sex | 0.99 | [0.87-1.12] | 0.842 |
|  | Race/ethnicity | 0.98 | [0.93-1.03] | 0.372 |
|  | Parental education | 1.01 | [0.85-1.20] | 0.880 |
|  | **Recreational cannabis legal status** | **0.78** | **[0.67-0.90]** | **<0.001^***^** |
|  | **Age** | **0.69** | **[0.63-0.76]** | **<0.001^***^** |
|  | **Total family income** | **0.80** | **[0.70-0.92]** | **<0.001^***^** |

*Notes*: The reference category for this model is Class 4 (moderate increasing), which represents the largest trajectory group with 3118 participants (41.5%). For predictors, the reference groups are as follows: female for biological sex, non-Hispanic white for race/ethnicity, high school education or less for parental education, non-legalized status for recreational cannabis laws, and total family income below $75,000. Statistical significance (* *p*<0.05, ** *p*<0.01, ****p*<0.001).

**Table S8.** Common effects of familial factors and psychopathological t-scores on positive expectancies of cannabis use effects across all trajectory classes

| Time | Variable | $\beta$ | SE | Est./SE | *p*-value |
| --- | --- | --- | --- | --- | --- |
| T1 | **Family cannabis use rules** | **-0.130** | **0.040** | **-3.274** | **0.001^**^** |
|  | **Parental monitoring** | **-0.089** | **0.040** | **-2.233** | **0.026^*^** |
|  | Family conflict | 0.018 | 0.010 | 1.843 | 0.065 |
|  | Psychopathology t-score | 0.001 | 0.002 | 0.703 | 0.482 |
| T2 | Family cannabis use rules | -0.132 | 0.068 | -1.938 | 0.053 |
|  | **Parental monitoring** | **-0.315** | **0.061** | **-5.193** | **<0.001^***^** |
|  | **Family conflict** | **0.061** | **0.016** | **3.843** | **<0.001^***^** |
|  | Psychopathology t-score | 0.002 | 0.003 | 0.729 | 0.466 |
| T3 | Family cannabis use rules | 0.025 | 0.052 | 0.475 | 0.635 |
|  | **Parental monitoring** | **-0.185** | **0.040** | **-4.563** | **<0.001^***^** |
|  | **Family conflict** | **0.068** | **0.010** | **6.514** | **<0.001^***^** |
|  | **Psychopathology t-score** | **0.004** | **0.002** | **2.201** | **0.028^*^** |

*Notes*: This table presents estimates from the common effects model, where predictor effects were constrained to be equal across all latent classes. The outcome variable is positive expectancies of cannabis use effects at each time point (T1–T3). Statistical significance (* *p*<0.05, ** *p*<0.01, ****p*<0.001).

**Table S9.** Correlation matrix for time-varying predictors

|  | Psychopathology | Parental monitor | Family cannabis use rules | Family conflict |
| --- | --- | --- | --- | --- |
| Time 1 |  |  |  |  |
| Psychopathology | 1 |  |  |  |
| Parental monitor | -0.14 | 1 |  |  |
| Family cannabis use rules | -0.05 | -0.01 | 1 |  |
| Family conflict | 0.17 | -0.29 | 0.04 | 1 |
|  |  |  |  |  |
| Time 2 |  |  |  |  |
| Psychopathology | 1 |  |  |  |
| Parental monitor | -0.16 | 1 |  |  |
| Family cannabis use rules | -0.04 | 0.02 | 1 |  |
| Family conflict | 0.17 | -0.25 | 0.00 | 1 |
|  |  |  |  |  |
| Time 3 |  |  |  |  |
| Psychopathology | 1 |  |  |  |
| Parental monitor | -0.14 | 1 |  |  |
| Family cannabis use rules | -0.04 | -0.01 | 1 |  |
| Family conflict | 0.19 | -0.26 | -0.01 | 1 |


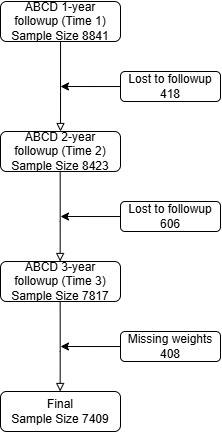
**Figure S1.** Flowchart of study participants
